# An Immune-CNS Axis Activates Remote Hippocampal Stem Cells Following Spinal Transection Injury

**DOI:** 10.3389/fnmol.2018.00443

**Published:** 2018-12-11

**Authors:** Sascha Dehler, Wilson Pak-Kin Lou, Liang Gao, Maxim Skabkin, Sabrina Dällenbach, Andreas Neumann, Ana Martin-Villalba

**Affiliations:** ^1^Molecular Neurobiology, German Cancer Research Center (DKFZ), Heidelberg, Germany; ^2^The Brain Cognition and Brain Disease Institute, ShenZhen Institutes of Advanced Technology, Chinese Academy of Sciences, Shenzhen, China

**Keywords:** neurogenesis, neural stem cells, hippocampus, spinal cord injuries, CD95/FAS

## Abstract

External stimuli such as injury, learning, or stress influence the production of neurons by neural stem cells (NSCs) in the adult mammalian brain. These external stimuli directly impact stem cell activity by influencing areas directly connected or in close proximity to the neurogenic niches of the adult brain. However, very little is known on how distant injuries affect NSC activation state. In this study, we demonstrate that a thoracic spinal transection injury activates the distally located hippocampal-NSCs. This activation leads to a transient increase production of neurons that functionally integrate to improve animal’s performance in hippocampal-related memory tasks. We further show that interferon-CD95 signaling is required to promote injury-mediated activation of remote NSCs. Thus, we identify an immune-CNS axis responsible for injury-mediated activation of remotely located NSCs.

## Introduction

The process of generating new neurons in the adult mouse brain is best characterized in the ventricular-subventricular zone (V-SVZ) and the subgranular zone (SGZ) of the dentate gyrus (DG). Neural stem cells (NSCs) within the V-SVZ generate neuronal precursors that migrate along the rostral migratory stream into the olfactory bulbs (OBs) where they disperse radially and generate functional interneurons that fine-tune odor discrimination. NSCs within the SGZ generate neuronal precursors that migrate short distance into the inner granule cell layer of the DG where they become functionally integrated into the existing network (Gage, 2000; Taupin and Gage, 2002; Zhao et al., 2008; Ming and Song, 2011; Aimone et al., 2014; Lim and Alvarez-Buylla, 2016). Hippocampal newborn neurons contribute to the formation of certain types of memories such as episodic and spatial memory (Kropff et al., 2015), as well as regulation of mood (Sahay and Hen, 2007) or stress (Snyder et al., 2011; Anacker et al., 2018). Adult neurogenesis is increased by various stimuli like an enriched environment, running and learning via neurotransmitters, hormones or growth factors (Kempermann et al., 1997, 2002; Nilsson et al., 1999; van Praag et al., 1999a,b; Shors et al., 2001; van Praag et al., 2005; Leuner et al., 2006; Lledo et al., 2006; Kobilo et al., 2011; Mustroph et al., 2012; Alvarez et al., 2016). In addition, endogenous NSCs can be activated by traumatic brain injury (Arvidsson et al., 2002; Parent et al., 2002; Thored et al., 2006; Hou et al., 2008; Liu et al., 2009).

In this study, we show that injury of the spinal cord transiently activates distantly located hippocampal stem cells. Some activated stem cells generate neurons in the hippocampal DG that transiently improve performance of injured mice in spatial memory and stress related tasks as compared to uninjured controls. Notably, we identify the interferon-gamma/CD95 signaling as necessary for activation of NSCs by a remote injury. In summary, our study unveils an immune-CNS interaction leading to injury-mediated activation of hippocampal neurogenesis.

## Materials and Methods

### Animals

**Figure 1:**
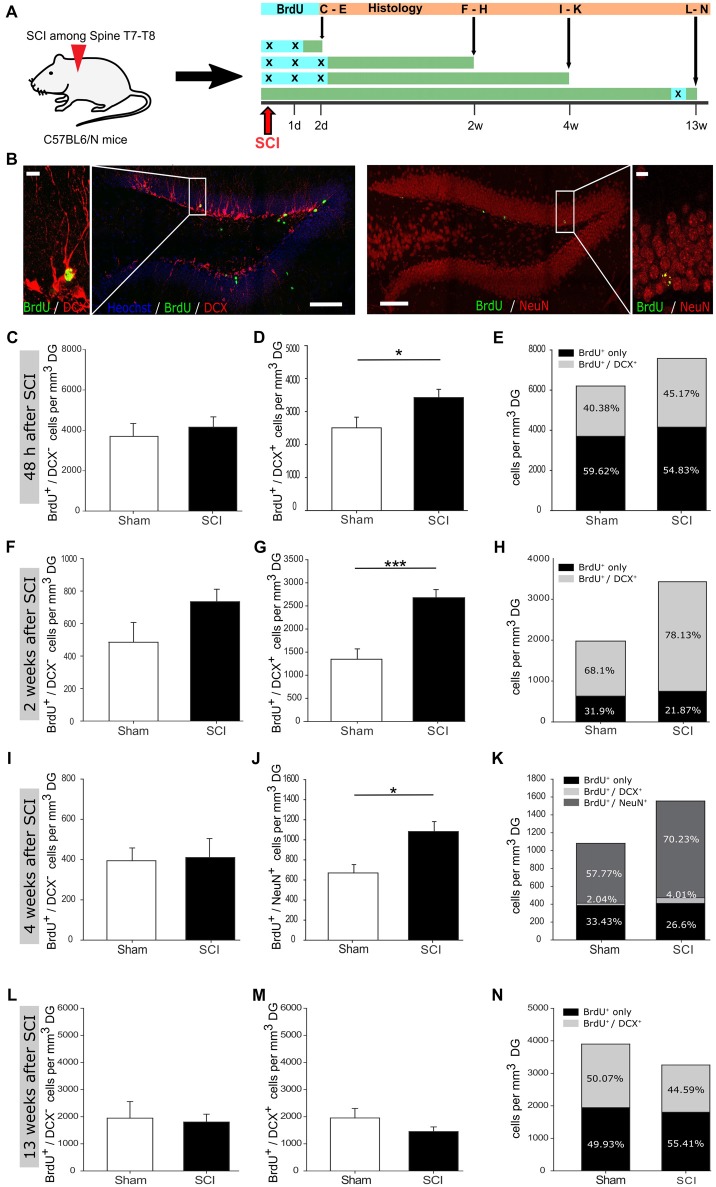
Increased hippocampal neurogenesis upon distant spinal cord injury. **(A)** Schematic illustration of the experimental timeline performed with C57BL/6N mice. **(B)** 5-Bromo-2-deoxyuridine (BrdU) incorporation within the dentate gyrus (DG) of adult mice; scale bar is 100 μm or 10 μm, respectively. **(C)** Quantification of BrdU^+^/DCX^−^ cells 48 h post injury in sham vs. SCI mice (3,698 ± 561 vs. 4,156 ± 434 cells/mm^3^ DG); *n*_sham_ = 6 vs. *n*_SCI_ = 6. **(D)** Quantification of BrdU^+^/DCX^+^ cells 48 h post injury, in sham vs. SCI mice (2,505 ± 323 vs. 3,422 ± 249 cells/mm^3^ DG); *n*_sham_ = 6 vs. *n*_SCI_ = 6. **(E)** Percentage distribution of BrdU^+^/DCX^+^ cells 48 h post injury in sham vs. SCI mice (40.38% vs. 45.17%). **(F)** Quantification of BrdU^+^/DCX^−^ cells 2 weeks post injury in sham vs. SCI mice (485 ± 109 vs. 734 ± 70 cells/mm^3^ DG); *n*_sham_ = 5 vs. *n*_SCI_ = 6. **(G)** Quantification of BrdU^+^/DCX^+^ cells 2 weeks post injury in sham vs. SCI mice (1,345 ± 224 vs. 2,677 ± 175 cells/mm^3^ DG); *n*_sham_ = 6 vs. *n*_SCI_ = 6. **(H)** Percentage distribution of BrdU^+^/DCX^+^ cells 2 weeks post injury in sham vs. SCI mice (68.1% vs. 78.13%). **(I)** Quantification of BrdU^+^/DCX^−^ cells 4 weeks post injury in sham vs. SCI mice (394 ± 57 vs. 410 ± 86 cells/mm^3^ DG); *n*_sham_ = 5 vs. *n*_SCI_ = 6. **(J)** Quantification of BrdU^+^/NeuN^+^ cells 4 weeks post injury in sham vs. SCI mice (669 ± 83 vs. 1,082 ± 99 cells/mm^3^ DG); *n*_sham_ = 6 vs. *n*_SCI_ = 6. **(K)** Percentage distribution 4 weeks post injury of BrdU^+^/NeuN^+^ cells in sham vs. SCI mice (57.77% vs. 70.23%) and BrdU^+^/DCX^+^ cells in sham vs. SCI (2.04% vs. 4.01%). **(L)** Quantification of BrdU^+^/DCX^−^ cells 13 weeks post injury in sham vs. SCI mice (1,947 ± 558 vs. 1,805 ± 270 cells/mm^3^ DG); *n*_sham_ = 6 vs. *n*_SCI_ = 8. **(M)** Quantification of BrdU^+^/DCX^+^ cells 13 weeks post injury in sham vs. SCI mice (2,005 ± 382 vs. 1,452 ± 159 cells/mm^3^ DG); *n*_sham_ = 5 vs. *n*_SCI_ = 8. **(N)** Percentage distribution of BrdU^+^/DCX^+^ cells 13 weeks post injury in sham vs. SCI mice (50.07% vs. 44.59%). All mice were 12 weeks old at the time of injury/sham-injury. Cell numbers are given as mean values (±SEM); *p < 0.05, ***p < 0.001; Student’s *t*-test.

For the experiments we used the following mouse lines: C57BL/6N, NesCreER^T2^CD95flox [B6.Cg-Tg(Nestin-Cre/Ers1)#GSc Fastm1Cgn] and IFNα-/IFNγ-R-KO [B6.Cg.Ifnar1tm1Agt Ifngr1tm1Agt/Agt]. Six weeks old NesCreER^T2^CD95flox (Cre^+^) and respective controls (Cre^−^) were intraperitoneally (i.p.) injected with 1 mg Tamoxifen (Sigma) twice a day for five consecutive days before operating. At the age of 12 weeks the respective group of mice received a sham or spinal transection injury as previously described (Letellier et al., 2010). For short term labeling of NSCs, mice received i.p. 5-bromo-2-deoxyuridine (BrdU; Sigma; 300 mg/kg bw) injections at 1 h, 24 h and 48 h post injury or a single shot injection 89 days post injury (Figures 1A, 4A,E), followed by a chase time of 1 day, 2 weeks or 4 weeks, respectively. For the long term label retaining experiment (Supplementary Figure S1A), 8-week-old mice received a daily single shot injection of BrdU (50 mg/kg bw) for a total duration of 3 weeks followed by a chase time of 17 weeks after the last BrdU injection. By giving a single shot of BrdU we were able to label a precise number of proliferating cells at a very specific time point. Multiple shots of BrdU were used to label enough amount of cells at the beginning, which would then allow having enough cells at the longer time points, and thus statistical power. The same BrdU-labeling protocol was used in (Seib et al., 2013), in which caspase-3 staining excluded any increase in apoptosis. For the isolation of primary NSCs, 8 weeks old C57BL/6N mice were used. All animals were housed in the animal facilities of the German Cancer Research Center (DKFZ) at a 12 h dark/light cycle with free access to food and water. For the injury and behavioral experiments, exclusively age-matched female mice were used. All animal experiments were performed in accordance with the institutional guidelines of the DKFZ and were approved by the “Regierungspräsidium Karlsruhe,” Germany.

**Figure 2:**
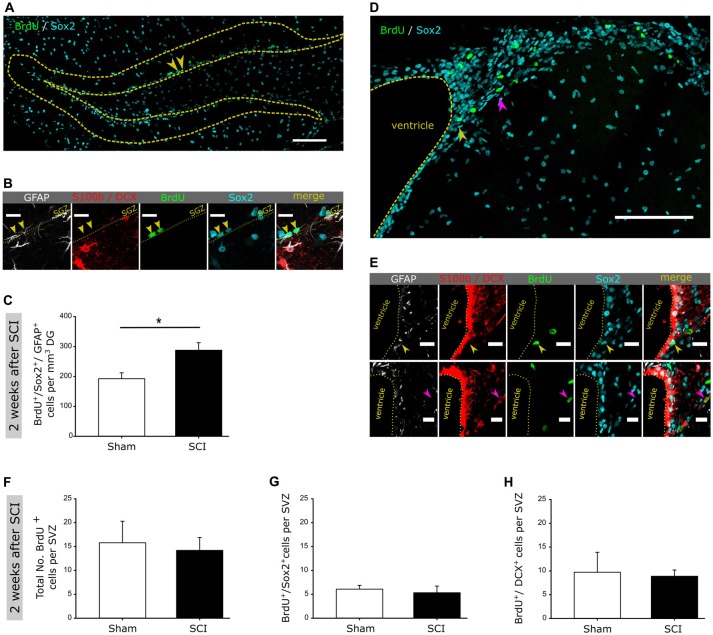
SCI does not affect neurogenesis within the ventricular-subventricular zone (V-SVZ). **(A)** Representative image of BrdU incorporation into Sox2 positive cells within the DG; scale bar is 100 μm. **(B)** Detailed image of a BrdU^+^/Sox2^+^/GFAP^+^ positive cell within the DG; scale bar is 15 μm. **(C)** Quantification of BrdU^+^/Sox2^+^/GFAP^+^ cells in the DG 2 weeks post injury in sham vs. SCI mice (192 ± 39 vs. 288 ± 50 cells/mm^3^ DG). **(D)** Representative image of BrdU incorporation into Sox2 positive cells within the V-SVZ; scale bar is 100 μm. **(E)** Detailed image of a BrdU^+^/Sox2^+^ and BrdU^+^/DCX^+^ cell within the V-SVZ; scale bar is 15 μm. **(F)** Quantification of the total number of BrdU^+^ cells in the V-SVZ 2 weeks post injury in sham vs. SCI mice (16 ± 5 vs. 14 ± 3 cells per SVZ). **(G)** Quantification of the total number of BrdU^+^/Sox2^+^ cells in the V-SVZ 2 weeks post injury in sham vs. SCI mice (6 ± 1 vs. 5 ± 2 cells per SVZ). **(H)** Quantification of the total number of BrdU^+^/DCX^+^ cells in the V-SVZ 2 weeks post injury in sham vs. SCI mice (10 ± 4 vs. 8 ± 1 cells per SVZ). All mice were 12 weeks old at the time of injury/sham-injury; group size *n*_naive_ = 4 vs. *n*_SCI_ = 4. Cell numbers are given as mean values (± SEM); *p < 0.05; Mann-Whitney Rank Sum Test.

**Figure 3:**
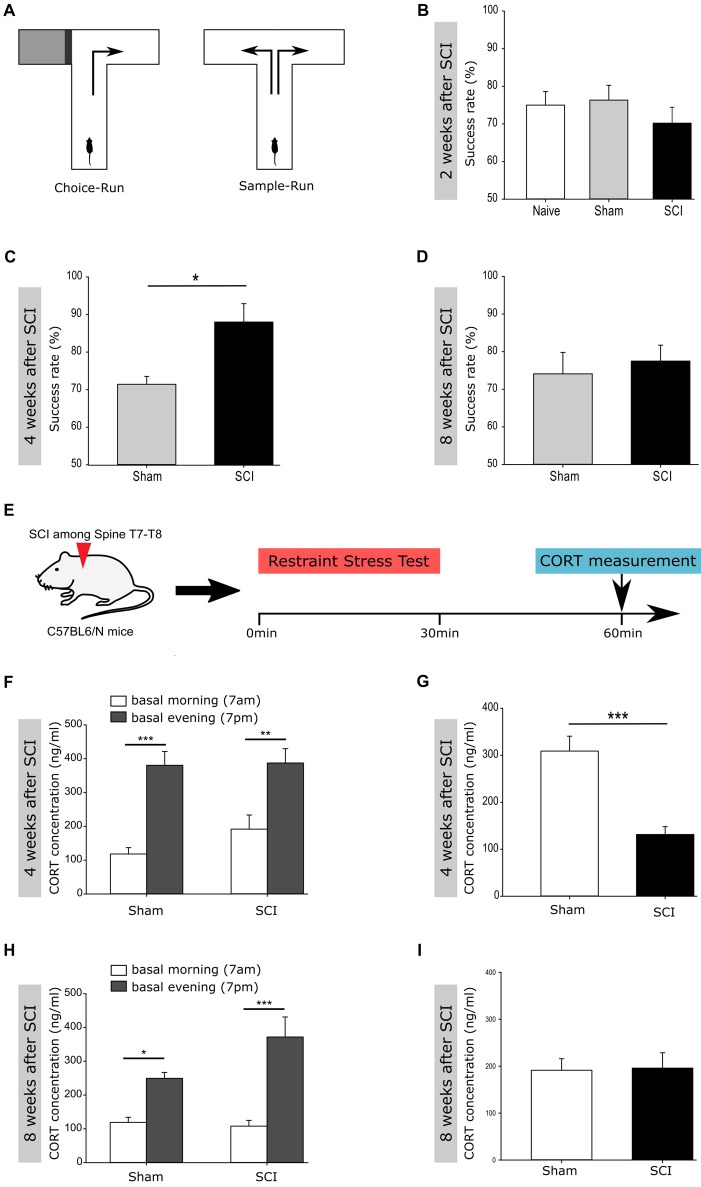
Improved performance in a Working Memory task and in buffering acute stress following spinal cord injury. **(A)** Experimental setup for the spontaneous alternation in the T-Maze test. **(B)** Success rate 2 weeks post injury of naïve vs. sham vs. SCI mice (75% ± 3.29 vs. 76.34% ± 3.80 vs. 70.19% ± 4.09); *n*_naive_ = 6 vs. *n*_sham_ = 14 vs. *n*_SCI_ = 13. **(C)** Success rate 4 weeks post injury of sham vs. SCI mice (71.43% ± 5.15 vs. 88% ± 4.38); group size, *n*_sham_ = 7 vs. *n*_SCI_ = 5; Mann-Whitney Rank Sum Test. **(D)** Success rate 8 weeks post injury of sham vs. SCI mice (74.11% ± 5.27 vs. 77.5% ± 3.79); group size, *n*_sham_ = 7 vs. *n*_SCI_ = 5. **(E)** Experimental setup to perform the restraint stress test. **(F)** Basal corticosterone (CORT) concentration 4 weeks post injury in sham_morning_ vs. sham_evening_ (118.2 ± 18.7 vs. 380.6 ± 40.9 ng/ml) and SCI_morning_ vs. SCI_evening_ (191.7 ± 42 vs. 387.5 ± 42.3 ng/ml); *n*_sham_ = 14 vs. *n*_SCI_ = 14; one way ANOVA. **(G)** CORT concentration 4 weeks post injury after the restraint stress test in sham vs. SCI mice (309 ± 31.6 vs. 131.3 ± 16.8 ng/ml); *n*_sham_ = 14 vs. *n*_SCI_ = 14; Mann-Whitney Rank Sum Test. **(H)** Basal CORT concentration 8 weeks post SCI in sham_morning_ vs. sham_evening_ (119 ± 15.2 vs. 249.5 ± 17.6 ng/ml) and SCI_morning_ vs. SCI_evening_ (108 ± 17 vs. 371.9 ± 59.1 ng/ml); *n*_sham_ = 13 vs. *n*_SCI_ = 13; one way ANOVA. **(I)** CORT concentration 8 weeks post SCI after the restraint stress test in sham vs. SCI mice (191.4 ± 24.6 vs. 196 ± 32.8 ng/ml); *n*_sham_ = 13 vs. *n*_SCI_ = 13. All mice were 12 weeks old at the time of injury/sham-injury. Succes rate and CORT concentration are given as mean values (±SEM); *p < 0.05, **p < 0.01, ***p < 0.001.

**Figure 4:**
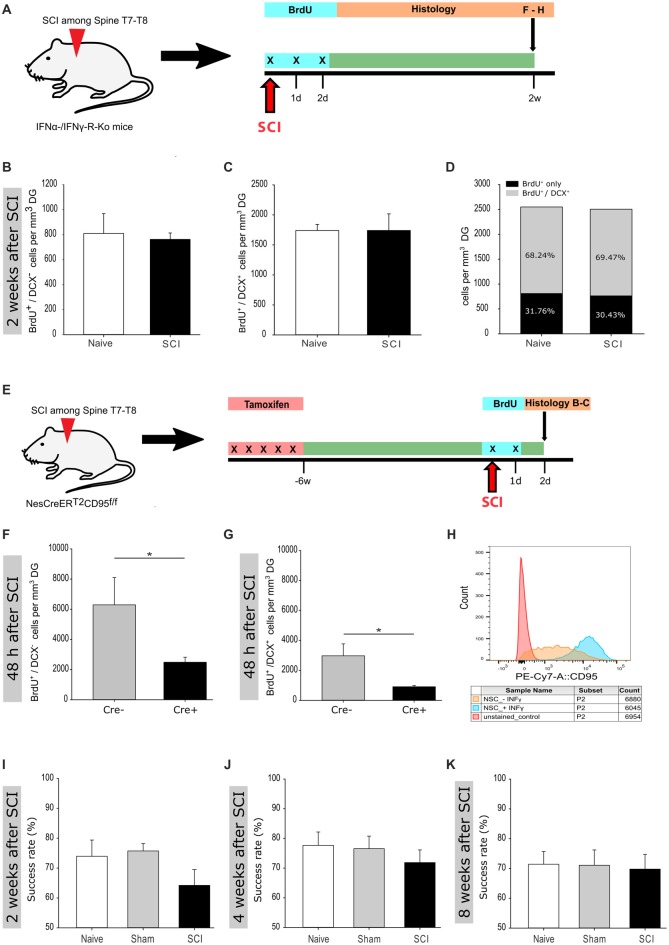
Reduced activation of adult hippocampal neurogenesis in IFNα-/IFNγ-R and CD95-Ko upon spinal cord injury. **(A)** Illustration of the experimental timeline performed with IFNα-/IFNγ-R-Ko mice. **(B)** Quantification of BrdU^+^/DCX^−^ cells 2 weeks post injury in naïve vs. SCI mice (809 ± 158 vs. 762 ± 51 cells/mm^3^ DG); *n*_naive_ = 4 vs. *n*_SCI_ = 5. **(C)** Quantification of BrdU^+^/DCX^+^ cells 2 weeks post injury in naïve vs. SCI mice (1,740 ± 101 vs. 1,741 ± 276 cells/mm^3^ DG); *n*_naive_ = 4 vs. *n*_SCI_ = 5. **(D)** Percentage distribution of BrdU^+^/DCX^+^ cells 2 weeks post injury in naïve vs. SCI mice (68.24 vs. 69.47%). **(E)** Illustration of the experimental timeline performed with NesCreER^T2^CD95^f/f^ mice. **(F)** Quantification of BrdU^+^/DCX^−^ cells 48 h post injury in injured Cre^−^ vs. injured Cre^+^ mice (6,292 ± 2,899 vs. 2,486 ± 662 cells/mm^3^ DG); $n_{Cre^{-}}=6$ vs. $n_{Cre^{+}}=6$; Student’s *t*-test. **(G)** Quantification of BrdU^+^/DCX^+^ cells 48 h post injury in injured Cre^−^ vs. injured Cre^+^ mice (2,976 ± 1,591 vs. 910 ± 157 cells/mm^3^ DG); $n_{Cre^{-}}=6$ vs. $n_{Cre^{+}}=6$; Student’s *t*-test. **(H)** Relative CD95 expression in unstained control, INFγ-untreated and -treated cells are illustrated in a single parameter histogram. **(I)** Success rate 2 weeks post injury of naïve vs. sham vs. SCI mice (73.96% ± 4.98 vs. 75.75% ± 2.33 vs. 64.22% ±4.62); *n*_naive_ = 6 vs. *n*_sham_ = 8 vs. *n*_SCI_ = 8. **(J)** Success rate 4 weeks post injury of naïve vs. sham vs. SCI mice (77.68% ± 4.49 vs. 76.56% ± 3.95 vs. 71.88 ± 3.98); *n*_naive_ = 7 vs. *n*_sham_ = 8 vs. *n*_SCI_ = 8. **(K)** Success rate 8 weeks post injury of naïve vs. sham vs. SCI mice (71.43% ± 4.28 vs. 71.09% ± 4.81 vs. 69.79 ± 3.91); *n*_naive_ = 7 vs. *n*_sham_ = 8 vs. *n*_SCI_ = 6. All mice were 12 weeks old at the time of injury/sham-injury. Cell numbers and success rate are given as mean (±SEM); *p < 0.05.

### Spinal Cord Injury

Female, age-matched animals were subjected to laminectomy at spine T7-T8 followed by a 80% transaction of the spinal cord injury by cutting the spinal cord with iridectomy scissors, as described in (Demjen et al., 2004; Stieltjes et al., 2006; Letellier et al., 2010). Sham mice were subjected only to laminectomy. Naïve mice did not face any surgical procedure.

### Handling of the Animals

Mice were habituated to the handling experimenter before starting with behavioral experiments. To this end, mice were handled for 5–10 min twice a day. Handling was performed for at least 5 days until the animals showed no anxiety-related behavior when meeting the experimenter.

### Spontaneous Alternation in the T-Maze

Spatial working memory performance was assessed on an elevated wooden T-Maze as described in (Corsini et al., 2009). Each animal had 4 sessions on the T-Maze (1 session/day; 4 trials/session). One trial consisted of a choice and a sample run. During the choice run one of the two target arms was blocked by a barrier according to a pseudorandom sequence, with equal numbers of left and right turns per session and with no more than two consecutive turns in the same direction. The mice were allowed to explore the accessible arm. Before the sample run (intertrial interval of ~10 s), the barrier was removed enabling accessibility to both arms. On the sample run the mouse was replaced back into the start arm facing the experimenter. The mouse was allowed to choose one of the two target arms. The trial was classified as success if the animal chose the previously blocked arm. For analysis all trials were combined and the success rate (%) was quantified [(# successful trials/# trials)*100].

### Restraint Stress Test

The mice were placed in a 50 ml canonical tube, equipped with a sufficient amount of breathing holes, for a duration of 30 min. Afterwards the mice were placed back into their housing cages for 30 min. Subsequently, blood samples of each mouse were isolated and the corticosterone (CORT) concentration was measured by using a CORT ELISA (IBL).

### Immunohistochemistry

Animals were sacrificed by using an overdose of Ketamin (120 mg/kg)/Xylazine (20 mg/kg) and were subsequently transcardially perfused with 20 ml 1× HBSS (Gibco) and 10 ml of 4% paraformaldehyde (Carl Roth). The brains were dissected and postfixed in 4% paraformaldehyde overnight at 4°C. A Leica VT1200 Vibratome was used to cut the tissue in 50 μm thick coronal sections. From each mouse six identical brain sections for DG and SVZ every 100 μm along the coronal axis were used for quantification. First, the brain sections were washed 3× 15 min at room temperature in TBS, followed by a 1 h blocking step in TBS^++^ (TBS with 0.3% horse serum (Millipore) and 0.25% Triton-X100 (Sigma)) at room temperature. Tissue was transferred to 0.5 ml Safe Lock Reaction-Tubes containing 200 μl TBS^++^ including primary antibodies. Samples were incubated for 24–48 h at 4°C. After incubating with primary antibody, tissue samples were washed 3× 15 min in TBS at room temperature, followed by a 30 min blocking step in TBS^++^ at room temperature. Brain sections were transferred to 0.5 ml Safe Lock Reaction-Tubes containing 200 μl TBS^++^ including secondary antibodies. Samples were incubated in the dark, for 2 h at room temperature. Finally the brain slices were washed 4× 10 min in TBS at room temperature, before they were further floated in 0.1M PB-Buffer and mounted on glass slides with Fluoromount G (eBioscience). The following antibodies were used: rat anti-BrdU (Abcam, 1/150), goat anti-DCX (Santa Cruz, 1/200), guinea pig anti-DCX (Merck, 1/400), rabbit anti-S100b (Abcam, 1/100), mouse anti-GFAP (Merck Millipore, 1/300), goat anti-Sox2 (Abcam, 1/200) and mouse anti-NeuN (Merck Millipore, 1/800). Nuclei were counterstained with Hoechst 33342 (Biotrend, 1/4,000).

### Microscopy and Cell Quantification

All images were acquired with a Leica TCS SP5 AOBS confocal microscope (Leica) equipped with a UV diode 405 nm laser, an argon multiline (458–514 nm) laser, a helium-neon 561 nm laser and a helium-neon 633 nm laser. Images were acquired as multichannel confocal stacks (Z-plane distance 2 μm) in 8-bit format by using a 20× (HCX PL FLUOTAR L NA0.40) oil immersion objective. Images were processed and analyzed in ImageJ (NIH). For representative images, the maximum intensity of a variable number of Z-planes was stacked, to generate the final Z-projections. Representative images were adjusted for brightness and contrast, applied to the entire image, cropped, transformed to RGB color format and assembled into figures with Inkscape. For cell quantification the entire volume of the DG was calculated by multiplying the entire area of the DG (middle plane of the total Z-stack) with the entire Z-stack size. The different cell populations were identified and counted (LOCI and Cell-Counter pug-in for ImageJ) based on their antibody labeling profile. Cell counts were either represented as cells/mm^3^ DG or as cells/DG.

### *In vitro* Culturing and Treatment of NSCs with INFγ

The lateral SVZ was microdissected as a whole mount as previously described (Mirzadeh et al., 2010). Tissue of one mouse was digested with trypsin and DNase according to the Neural Tissue Dissociation Kit (Miltenyi Biotec) in a Gentle MACS Dissociator (Miltenyi Biotec). Cells were cultured and expanded for 8–12 days in Neurobasal medium (Gibco) supplemented with B27 (Gibco), Heparine (Sigma), Glutamine (Gibco), Pen/Strep (Gibco), EGF (PromoKine) and FGF (PeloBiotech) as used in (Walker and Kempermann, 2014). For stimulation with INFγ (Millipore), 4 × 10^5^ cells were seeded. The next day, cells were treated with 50 ng INFγ/ml media for duration of 14 h.

### Flow Cytometric Analysis

The cells were harvested and were treated with Accutase (Sigma) for 5 min at 37°C, followed by filtering the cells with a 40 μm cell strainer to get a single cell suspension. Afterwards the cells were washed twice with FACS media (PBS/10%FCS) and were re-suspend in 200 μl FACS media. Cells were stained for 30 min at room temperature by using the Jo2 CD95::PECy7 antibody (BD Pharming/ 1/100). Afterwards the cells were washed three times with FACS media and were finally re-suspend in 200 μl FACS media.

### Statistics

Statistical analysis was performed with SigmaPlot Student’s *T*-Test, Mann-Whitney Rank Sum Test and one sided ANOVA. The respective statistical analysis as well as *p*-values is indicated in figure legends.

## Results

### Distinct Activation of Hippocampal Neurogenesis Following Spinal Cord Injury

To assess whether a remote CNS injury would activate NSCs in the SGZ, we injured the spinal cord at thoracic level T7-T8. In order to detect the reaction of SGZ-NSCs and their neurogenic progeny, we labeled these cells with BrdU (once daily) at the time of injury and in the following 24 h, 48 h or after 89 days and examined them at 2 days, 2 weeks, 4 weeks and 13 weeks following injury (Figure 1A). Brains were stained for BrdU, to follow actively dividing NSCs and transient amplifying progenitors (TAPs) cells on their transition to BrdU^+^/DCX^+^ neuroblasts and BrdU^+^/NeuN^+^ newborn neurons (Figure 1B). Already 48 h after injury, we observed a significant increase in new-born neuroblasts (Figures 1C–E). Two weeks following injury, the number of neuroblasts remained significantly higher when compared to sham-injured controls (Figure 1G). The population of BrdU^+^/DCX^−^ that encompass NSCs and TAPs showed a clear trend towards higher numbers in injured compared to sham-operated mice (Figures 1F,H). We therefore, proceed to specifically addressed NSCs in the DG 2 weeks after injury by staining for BrdU and the NSC specific markers Sox2 and GFAP as well as the astrocyte marker S100b and neuroblast marker DCX (Figures 2A,B). The number of BrdU^+^/Sox2^+^/GFAP^+^ NSCs was significantly increased in spinal cord injured mice as compared to sham controls (Figure 2C). We further assessed the maturation of BrdU-labeled cells to neurons (BrdU^+^/NeuN^+^) at 4 weeks after the injury. Significantly more newborn neurons were identified in the DG of the injured mice, whereas the number of BrdU^+^/DCX^−^ cells was comparable in injured and sham controls (Figures 1I–K). This 61.7% increase in newborn neurons is surprisingly high, since already an increase of 13% of newborn DG-neurons in the aging hippocampus through increased Wnt activity dramatically improved the performance of the animals in hippocampal-dependent memory tasks (Seib et al., 2013). Notably, at 13 weeks following injury, the number of cycling BrdU^+^/DCX^−^ cells and newborn neuroblasts was set back to basal levels, exhibiting similar numbers to that of its sham operated counterparts (Figures 1L–N).

Injury has been shown to activate a pool of highly dormant cells in the hematopoietic system (Wilson et al., 2008; Essers et al., 2009; Essers and Trumpp, 2010). To test if this is also the case for SGZ-NSCs, we used a 3 weeks BrdU-labeling protocol starting at the age of 8 weeks and allowed a chase time of 16 weeks after the last BrdU injection. Mice were subjected to spinal cord injury at 14 weeks chase time or left uninjured and sacrificed 2 weeks later to follow the reaction to injury of the highly dormant NSCs (Supplementary Figure S1A). Notably, the number of BrdU^+^ cells in the DG was significantly reduced in injured mice as compared to sham controls (Supplementary Figures S1B,C). Since, it is known that a local injury to the brain activates the migration of NSCs in close vicinity out of the neurogenic niche (Nakatomi et al., 2002; Grande et al., 2013), we assessed a potential migration of BrdU-labeled cells to the neighboring regions of the fimbria-fornix (FF) and corpus callosum (CC; Supplementary Figure S1B). The number of BrdU-labeled cells in FF and CC regions was higher in injured than naïve counterparts (Supplementary Figures S1D,E). In summary our data suggest that spinal cord injury activates local neurogenesis within the SGZ of the DG, and reduces the fraction of a dormant label-retaining cells within the SGZ. Further studies shall further address the nature of these label retaining cells and whether they migrate out to nearby regions.

We next assess whether spinal cord injury would also activate neuronal production in NSCs within the other V-SVZ neurogenic niche (Figures 2D–H). Notably, the total numbers of BrdU-labeled cells, BrdU-NSCs or neuroblasts were comparable in sham and SCI animals (Figures 2F–H). Together, our data shows that distant spinal cord injury stimulates a fast but transient activation of NSCs residing in the remote SGZ of the DG to generate neurons, but not in the V-SVZ of the lateral ventricles. How a distant injury specifically affects local hippocampal NSCs through interferons will be subject of future studies.

### Spinal Cord Injury Leads to Better Working Memory and an Improved Buffering of Acute Stress

Together, we see that the injury activates both, normal homeostatic neurogenesis and decreases the pool of highly dormant stem cells potentially by activating their migration out of the DG. Therefore, we next tested the function of the injury-induced surplus of newborn neurons within the hippocampus of injured mice, homeostatic neurogenesis. Adult hippocampal neurogenesis has been shown to positively impact short- and long-term spatial working memory, navigation learning, pattern discrimination as well as trace and contextual fear conditioning (Corsini et al., 2009; Deng et al., 2010; Aimone et al., 2011), but also to counteract depression- and stress-induced behavioral responses (Sahay and Hen, 2007; Snyder et al., 2011). To test the function of injury-induced newborn neurons in the DG, we tested the performance of injured and naïve mice in a hippocampal-dependent task, the spontaneous alternation on an elevated T-Maze, used as readout of short term spatial working memory (Figure 3A). Even if spinal cord injured mice definitely experience motor dysfunctions, no differences between the injured and sham-injured group were detected in terms of reaction time/decision time while performing the elevated T-Maze test. Mice were tested at 2, 4 and 8 weeks following spinal cord injury. At 2 weeks post-injury naïve, sham and spinal cord injured mice showed a similar success rate of the spontaneous alternation (Figure 3B). Importantly, at 4 weeks following injury, the success rate of injured mice was significantly higher than the rate of Sham controls (Figure 3C). Notably, the improved performance of injured mice on the T-Maze disappeared at 8 weeks post-injury (Figure 3D).

Another reported function of newborn DG-neurons is buffering of acute stress, which would be very beneficial following injury (Snyder et al., 2011). To test the behavioral response to an acute stress situation, we performed a restraint stress test (Figure 3E) at 4 and 8 weeks following spinal cord or sham-injury. CORT, a corticosteroid that is produced in the cortex of the adrenal glands and released into the blood stream, is classically used as readout for various stress situations in rodents (Gong et al., 2015). The basal CORT concentration within the bloodstream of rodents is increased during daytime under homeostatic conditions, as shown in the tested groups before the stress test (Figures 3F,H). At 4 weeks post-injury the injured mice showed significantly lower levels of CORT in the bloodstream as compared to the sham-injured mice (Figure 3G). Notably, at 8 weeks following injury the CORT levels exhibit similar blood concentrations following restraint stress in both experimental groups (Figure 3I).

Taken together, our data demonstrated that newly generated neurons integrate into the existing hippocampal network and positively influence the performance of injured mice in a hippocampal-dependent spatial memory task and in buffering acute stress situations. However, as the observed activation of neurogenesis, the functional improvement is also transient. Interestingly, we previously observed a transient increase in neurogenesis following exercise that improved performance on the T-Maze in an equally transient mode (Corsini et al., 2009). Thus, our data suggest that newborn functionally immature neurons impact short term memory and the buffering of acute stress as long as they are young and plastic. However, this effect disappears as they become similar to their older counterparts (Kropff et al., 2015).

### Loss of IFNα-/IFNγ-R and CD95 Inhibits Neural Stem Cell Activation Upon Spinal Transection Injury

Acute tissue injury activates an immediate inflammatory response that is able to rapidly affect distant locations. Notably, we previously identified interferons as an activator of NSCs in the V-SVZ following a global ischemic insult that induces damage in the nearby located striatum (Llorens-Bobadilla et al., 2015). The requirement of IFNγ signaling for SCI-mediated activation of SGZ-NSCs was further tested using mice deficient in IFNα-/IFNγ-receptor (Figure 4A) as compared to wt counterparts (Figures 1F–H). Excitingly, 2 weeks following injury, IFNα-/IFNγ-receptor deficient mice did neither show a significant increase in the population of neuroblasts (BrdU^+^/DCX^+^), nor in the population of BrdU^+^/DCX^−^ cells (Figures 4B–D). These observations indicated that spinal transection injury does not activate SGZ-NSCs lacking a functional IFNα/IFNγ-signaling-pathway. We next investigated the putative signaling pathways involved in local SCI-mediated activation of SGZ-NSCs. Interferons have been reported to increase the expression of CD95-ligand and CD95 (Chow et al., 2000; Kirchhoff et al., 2002; Boselli et al., 2007). In a previous study we demonstrated that the TNF-R family member, CD95, is required for the activation of SGZ-NSCs following global ischemia (Corsini et al., 2009). To test the regulation of CD95 upon IFNγ treatment in NSCs, we isolated NSCs from the V-SVZ of 8 weeks old C57BL/6N mice, cultured them *in vitro* for short time and exposed them for 14 h to IFNγ. Thereafter expression of CD95 was analyzed by Flow Cytometry. IFNγ significantly increased the expression of CD95 in NSCs as compared to untreated NSCs (Figure 4H and Supplementary Figure S2). To assess CD95’s involvement in SCI-induced neurogenesis we used the NesCreER^T2^CD95^f/f^ mouse line. This mouse line enables an acute deletion of CD95 in the adult NSC compartment (Corsini et al., 2009). CD95NesCreER^T2+^ (Cre^+^) and CD95NesCreER^T2-^ (Cre^−^) mice received tamoxifen injections at 6 weeks of age. Their spinal cord was injured at the age of 12 weeks. Dividing cells were labeled by BrdU at the time of injury and 24 h post injury. The SGZ was further processed for staining of BrdU and DCX 48 h after the surgery (Figure 4E). CD95-deficient NSCs exhibit an impaired injury-induced activation, as significantly fewer BrdU^+^/DCX^−^cells and newborn neuroblasts (BrdU^+^/DCX^+^) could be detected in the SGZ of Cre^+^ mice as compared to their injured Cre^−^ counterparts (Figures 4F,G). Thus, CD95 is locally involved in activation of SGZ-NSCs by a remote injury. Next, we set out to test if the injury-induced improvement of the spatial working-memory is due to the increased activation of NSCs. Indeed, injured and sham operated IFNα-/IFNγ-receptor deficient mice showed a similar success rate in the spontaneous alternation in the elevated T-Maze (Figures 4I–K). Thus, interferon-related increase of homeostatic neurogenesis mediates the functional improvement in short-term working memory exhibited by spinal injured animals. Altogether, our results indicate that injury-induced IFN signaling triggers CD95 activation of SGZ-NSCs, thereby leading to a transient expansion of the pool of newborn neurons resulting in an improved working memory.

## Discussion

Here, we examine how a remote injury to the CNS influences distally located SGZ-NSCs, short and long term post-injury. Our data clearly show an acute and transiently increased activation of adult SGZ-NSCs to produce neurons following a remote injury and suggest that a fraction of highly dormant stem cells are activated to migrate out of the neurogenic niche. Notably, we show that the newly generated neurons functionally integrate into the existing network, as demonstrated in an elevated spatial navigation performance, spontaneous alternations on a T-Maze test, which provides a very sensitive test to detect dysfunction of the hippocampus in rodents (Deacon and Rawlins, 2006; Zhang et al., 2004). In addition, SCI mice exhibited a higher ability to buffer acute restraint stress than control counterparts, consistent with the previously reported role of hippocampal neurogenesis in regulating the hypothalamic-pituitary-adrenal axis (Snyder et al., 2011).

However, this activation of neurogenesis fades away with time. Accordingly, two studies investigated the effects of spinal cord injury to the neurogenic niches in adult *Sprague-Dawley* rats and detected a decreased level of adult V-SVZ and SGZ neurogenesis 60 days post spinal cord injury (Felix et al., 2012; Jure et al., 2017). Besides, studies of hematopoietic stem cell (HSC) activation by inflammatory signals, show that an acute exposure activates the quiescent population of HSCs, whereas chronic exposure negatively impact HSC activation (Essers et al., 2009).

As already hypothesized by Felix et al. (2012) and in line with Essers et al. (2009), we show that inflammatory signatures, released in an acute phase post spinal cord injury, play a major role in transmitting the injury signal towards the hippocampus to activate adult neurogenesis. It is known that an injury to the spinal cord would activate a multiphase immune response including macrophage/microglia activation within the spinal cord (Letellier et al., 2010; Abdanipour et al., 2013). Using single cell transcriptomics we identified activation of an interferon-gamma-response in NSCs as necessary for injury-induced activation of V-SVZ NSCs in a model of global ischemia (Llorens-Bobadilla et al., 2015). However, also in this setting we could not detect expression of the interferon transcripts in stem cells or niche cells, other than microglia. Interferon mRNAs are very low abundant and thus hardly to detect in most non-immune cells. In the aging brain interferon mRNA was detected in the plexus choroideus in rodents and humans (Baruch et al., 2014). We can only speculate that in the case of stroke and of a distant spinal injury local microglia, plexus choroideus or systemic signals provide the upstream regulator of the interferon-response in NSCs.

In the current study, we identify interferons as the main factor that transmits the injury signal from the spinal cord towards the hippocampus, where through activation of CD95 stem cells exit the quiescent state to differentiate into neurons. This results are consistent with the previously reported role of CD95 in activation of hippocampal NSCs following a global ischemic injury (Corsini et al., 2009). In NSCs CD95 does not induce apoptosis, but on the contrary, increases their survival and differentiation. Notably, transplantation of CD95-activated NSCs, but not of control non treated counterparts, rescued the hippocampal-related memory deficits following global ischemia (Corsini et al., 2009). This study also showed that CD95-deficient mice show a reduced basal level of neurogenesis within the DG. However, running activity was able to activate CD95-deficient NSCs within the DG, which makes it even more remarkable, that SCI fails to do so (Corsini et al., 2009). Expression of CD95-ligand increased following ischemic injury to the brain in humans and rodents. In a follow up study, characterizing the single cell transcriptomes of NSCs in the ischemic SVZ, we detected an injury-induced increase of cells expressing CD95 transcripts, that was lower in interferon-receptor deficient mice (Llorens-Bobadilla et al., 2015).

The observed transition from a quiescent to an active state, triggered by a distant injury site, in effects seems to be similar to the transition from G_0_ to an elevated G_alert_ state in muscle satellite cells (Rodgers et al., 2014). Interestingly, this alert state is triggered in distant stem cells in contralateral muscles, and is also observed in other tissue stem cells such as HSCs (Rodgers et al., 2014). Stem cells in an alert state are primed for cell cycle entry to react in a much faster and efficient way to incoming injuries of different nature. Here, we show that a remote CNS injury triggers different responses in actively dividing and dormant NSCs. While actively dividing NSCs are engaged in homeostasis, the fraction of dormant cells decreases, presumably to take potential alternative migratory pathways to injury-associated areas. Of course, local activation and proliferation of glia cells might play a role and should not be underestimated, but these astrocytes would have been proliferating at the time of labeling and only become reactivated by local brain injury, which is a less probable behavior. However, future studies are needed to follow up the fate of these highly dormant stem cells.

What could be the role of an increased production of granule cell neurons in the hippocampus? Certainly, spinal cord injury represents a very stressful state for the whole organism. It has been shown that adult hippocampal neurogenesis is on the one hand strongly influenced by chronic and acute stress (Conrad et al., 1999; Kirby et al., 2013; LaDage, 2015), on the other hand increased neurogenesis ameliorates stress (Snyder et al., 2011; Anacker et al., 2018). We show that spinal cord injury buffers acute restraint stress, and this buffering disappears when active neurogenesis does. Thus, we hypothesized that injury-induced neurogenesis buffers stress and thereby improves behavioral adaptation to the post-traumatic situation.

In summary, our data show that an acute injury to the spinal cord activates hippocampal neurogenesis, resulting in a transiently increased production of newborn neurons that are functional, as shown by the improved performance in spatial memory tasks of injured mice. Furthermore, we identified interferons as a major factor involved in activation via CD95 of distant stem cells.

## Author Contributions

SDe performed experiments involving cell counts, interferon, stress, behavioral read out following spinal injury, analyzed and interpreted data, and wrote the manuscript. WP-KL and LG performed spinal injuries. MS: experiments related to interferon in stem cell cultures. SDä and AN: cell counts following spinal injuries in wt and CD95ko mice, analyzed and interpreted related data. AM-V: project design and oversight, data interpretation, and wrote the manuscript.

## Conflict of Interest Statement

The authors declare that the research was conducted in the absence of any commercial or financial relationships that could be construed as a potential conflict of interest. The reviewer SDM and handling editor declared their shared affiliation at time of review.

## Supplementary Material

FIGURE S1 Dormant NSCs are activated following spinal transection injury. **(A)** Schematic illustration of the experimental timeline for labeling dormant NSCs in the DG of adult C57BL/6N mice. **(B)** Representative coronal section of the adult mouse brain with designated regions for the quantification of BrdU^+^ labeled cells. **(C)** Quantification of BrdU^+^ labeled cells in the DG in sham vs. SCI mice (11,437 ± 1,255 vs. 7,532 ± 1,017 cells/mm^3^ DG); *n*_sham_ = 5 vs. *n*_SCI_ = 11. **(D)** Quantification of BrdU^+^ labeled cells in the FF in naïve vs. SCI mice (4,010 ± 913 vs. 6,326 ± 906 cells/mm^3^ FF); *n*_naive_ = 4 vs. *n*_SCI_ = 6. **(E)** Quantification of BrdU^+^ labeled cells in the CC in naïve vs. SCI mice (1,848 ± 665 vs. 7,210 ± 2,829 cells/mm^3^ CC); *n*_naive_ = 4 vs. *n*_SCI_ = 6. All mice were 28 weeks old at the time of injury/sham-injury. Cell numbers are given as mean (±SEM); **p* < 0.05; Student’s *t*-test.

FIGURE S2 Related to Figure 4. Strategy to determine relative CD95 expression in cultured NSCs by using Flow Cytometry. First gate uses FSC/SSC gating to exclude cellular debris; second gate excludes cell aggregates and third shows relative CD95 expression in unstained control cells **(A)**, stained IFNγ-untreated cells **(B)** and stained IFNγ-treated cells **(C)**.
