# Supplementary figures and images for: An Immune-CNS Axis Activates Remote Hippocampal Stem Cells Following Spinal Transection Injury
Source: Front Mol Neurosci. 2018 Dec 11;11:443. doi: 10.3389/fnmol.2018.00443 (PMC6299844; doi:10.3389/fnmol.2018.00443)

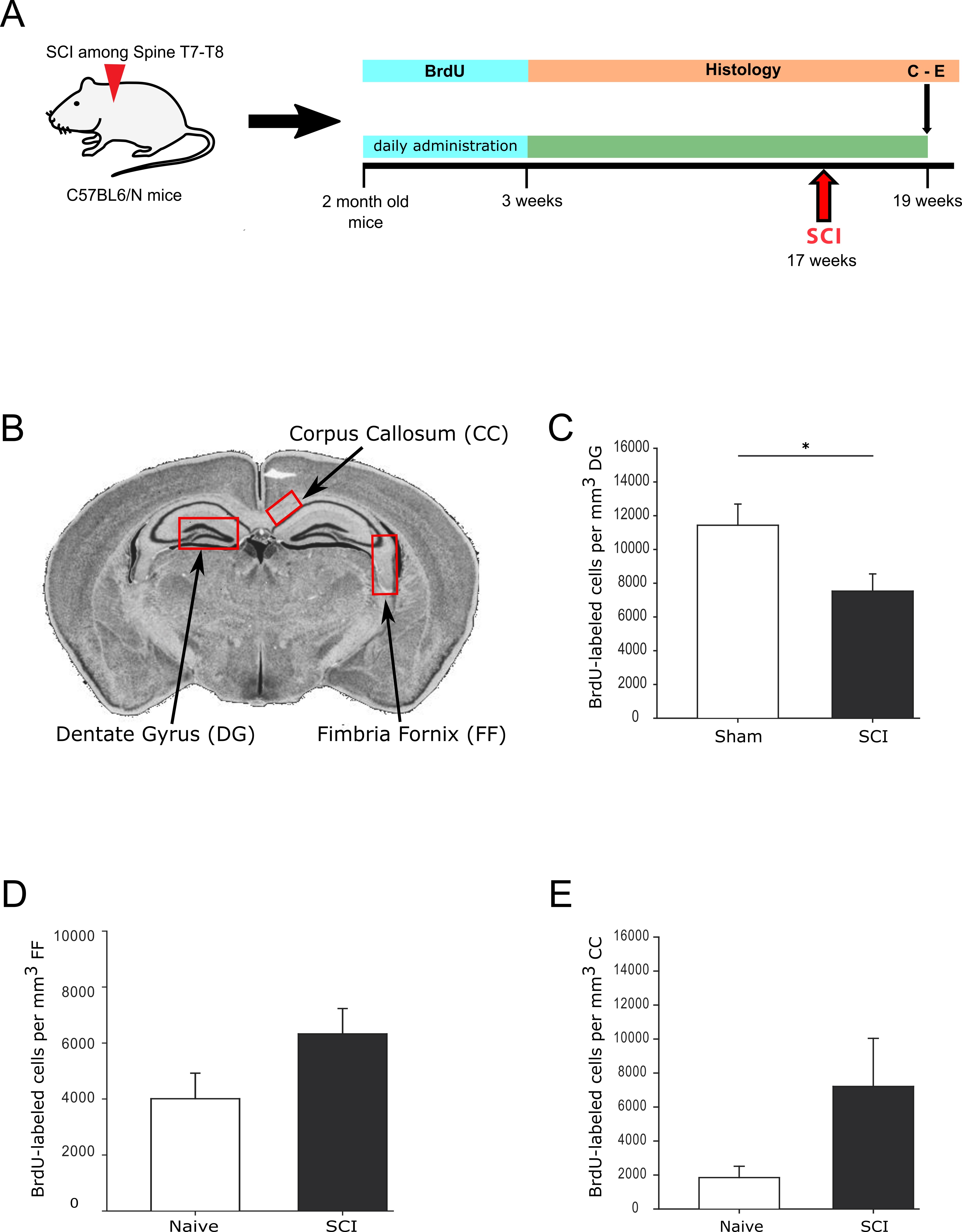

Supplement: FIGURE S1 — Dormant NSCs are activated following spinal transection injury. (A) Schematic illustration of the experimental timeline for labeling dormant NSCs in the DG of adult C57BL/6N mice. (B) Representative coronal section of the adult mouse brain with designated regions for the quantification of BrdU+ labeled cells. (C) Quantification of BrdU+ labeled cells in the DG in sham vs. SCI mice (11,437 ± 1,255 vs. 7,532 ± 1,017 cells/mm3 DG); nsham = 5 vs. nSCI = 11. (D) Quantification of BrdU+ labeled cells in the FF in naïve vs. SCI mice (4,010 ± 913 vs. 6,326 ± 906 cells/mm3 FF); nnaive = 4 vs. nSCI = 6. (E) Quantification of BrdU+ labeled cells in the CC in naïve vs. SCI mice (1,848 ± 665 vs. 7,210 ± 2,829 cells/mm3 CC); nnaive = 4 vs. nSCI = 6. All mice were 28 weeks old at the time of injury/sham-injury. Cell numbers are given as mean (±SEM); *p < 0.05; Student’s t-test. [file Image_1.JPEG]

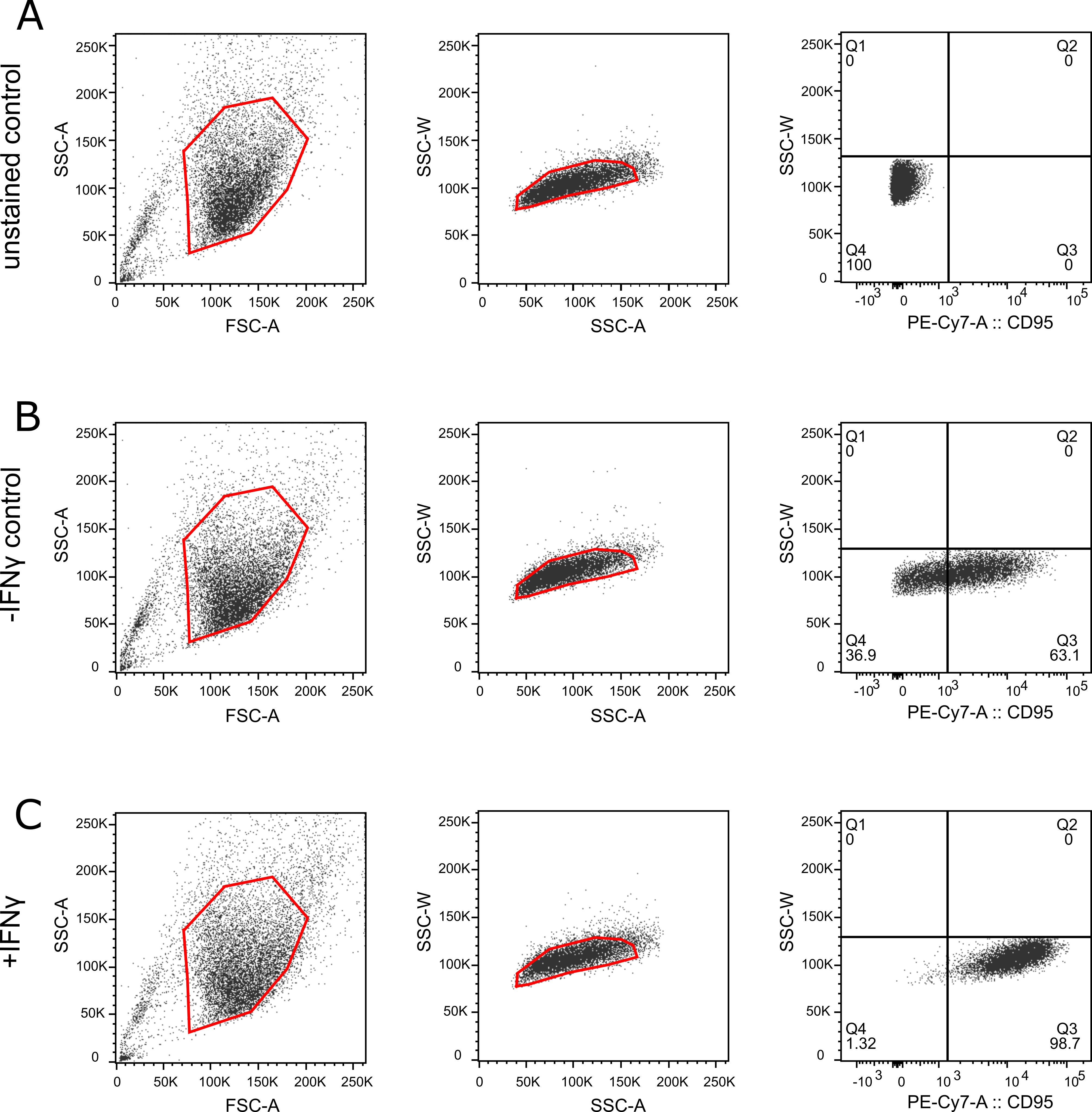

Supplement: FIGURE S2 — Related to Figure 4. Strategy to determine relative CD95 expression in cultured NSCs by using Flow Cytometry. First gate uses FSC/SSC gating to exclude cellular debris; second gate excludes cell aggregates and third shows relative CD95 expression in unstained control cells (A), stained IFNγ-untreated cells (B) and stained IFNγ-treated cells (C). [file Image_2.JPEG]
